# Development of an optogenetic gene sensitive to daylight and its implications in vision restoration

**DOI:** 10.1038/s41536-021-00177-5

**Published:** 2021-10-14

**Authors:** Yoshito Watanabe, Eriko Sugano, Kitako Tabata, Akito Hatakeyama, Tetsuya Sakajiri, Tomokazu Fukuda, Taku Ozaki, Tomoya Suzuki, Tatsuki Sayama, Hiroshi Tomita

**Affiliations:** 1 Graduate Course in Biological Sciences, Iwate University Division of Science and Engineering, 4-3-5 Ueda, Morioka, Iwate 020-8551 Japan; 2 Department of Nutrition, Kyushu Nutrition Welfare University, 5-1-1 Shimoitozu, Kitakyushu Kokurakita-Ku, Fukuoka, 803-0846 Japan; 3 Clinical Research, Innovation and Education Center, Tohoku University Hospital, 1-1 Seiryo, Aoba, Sendai, Miyagi 980-8574 Japan

**Keywords:** Cell death in the nervous system, Retina

## Abstract

Optogenetic gene-mediated therapy for restoring vision is thought to be a useful treatment for blind patients. However, light sensitivity achieved using this gene therapy is inferior to that of daylight vision. To increase light sensitivity, we designed three mutants using a bioinformatics approach. Nucleotide sequences encoding two sites in the extracellular loops (ex1, ex3) of mVChR1 close to simulated ion-conducting pathways were replaced by homologous amino acid-encoding sequences of ChR1 or ChR2. The light sensitivity of ex3mV1 was higher than that of mVChR1 at 405–617 nm. Visual responses were restored in Royal College of Surgeons rats with genetically degenerating photoreceptor cells transfected with ex3mV1Co, wherein transmembrane of sixth (TM6) in ex3mV1 was additionally replaced with the corresponding domain of CoChR; these rats responded to light in the order of μW/mm^2^. Thus, ex3mV1Co might be useful for the restoration of advanced visual function.

## Introduction

The light-driven cation channel channelrhodopsin-2 (ChR2)^1,2^ plays a central role in optogenetics. Optogenetic techniques have been used in a variety of fields. In neuroscience studies, the *ChR2* gene has been transfected into neuronal cells for the optical control of neural^3^ and behavioural^4^ activities and for performing studies on brain function^5^ and mechanisms of neuronal diseases^6,7^. In the field of vision research, optogenetic techniques have been used to treat blindness^8^. Bi et al.^9^ and our group^10–12^ reported that the transfection of *ChR2* into the retinal ganglion cells (RGCs) of blind mice and rats could recover their light responses. Safety studies have also been performed in rats^13^ and marmosets;^14^ continuous expressions of the *ChR2* gene derived from the unicellular green alga *Chlamydomonas reinhardtii* did not result in any adverse effect caused by immunological responses. Currently, a few clinical trials (NCT02556736, NCT03326336^15^) are underway for investigating the efficacy of gene therapies employing optogenetic genes for restoring vision.

Previously, we had developed modified Volvox channelrhodopsin-1 (*mVChR1*), which has a broader range of wavelength sensitivities than that of ChR2^16^. We found that the transfection of the *mVChR1* gene into blind rats restored their VEPs upon stimulation at 450–600 nm and that optomotor responses were elicited with all colour stripes. We also found that the recovered visual responses were maintained over a year without any adverse effects^17^. However, unnaturally bright light intensities were required to elicit responses. We also reported that the dual expression of optogenetic genes in the retina expands the range of wavelength sensitivities but not that of light sensitivities^18^. This motivated us to develop a channelrhodopsin that is more photosensitive than mVChR1.

Various types of channelrhodopsins have been developed, such as ChR2 (E123T/T159C)^19^ and ChIEF (chimera)^20^. However, most of them have been evaluated in experiments that utilized light in the order of mW/mm^2^ for stimulation. Because daylight is dimmer (μW/mm^2^ rather than mW/mm^2^), channelrhodopsins should have higher light sensitivities or at least be responsive to light in the order of μW/mm^2^ for restoring vision in blind patients.

Here, we report the development of multiple μW/mm^2^ light-responsive channelrhodopsins by the replacement of amino acids related to ion-conducting pathways in mVChR1 with corresponding ones from ChR2. We also characterized the photosensitivity of the modified channelrhodopsin ex3mV1Co by patch–clamp recording in the human embryonic kidney (HEK) 293 cells and in vivo experiments using genetically blind rats. Our newly developed optogenetic gene, with its higher and broad-spectrum light sensitivities, would be especially useful for gene therapy aimed at restoring daylight vision.

## Results

### Design of mutants by bioinformatics approaches

**Fig. 1:**
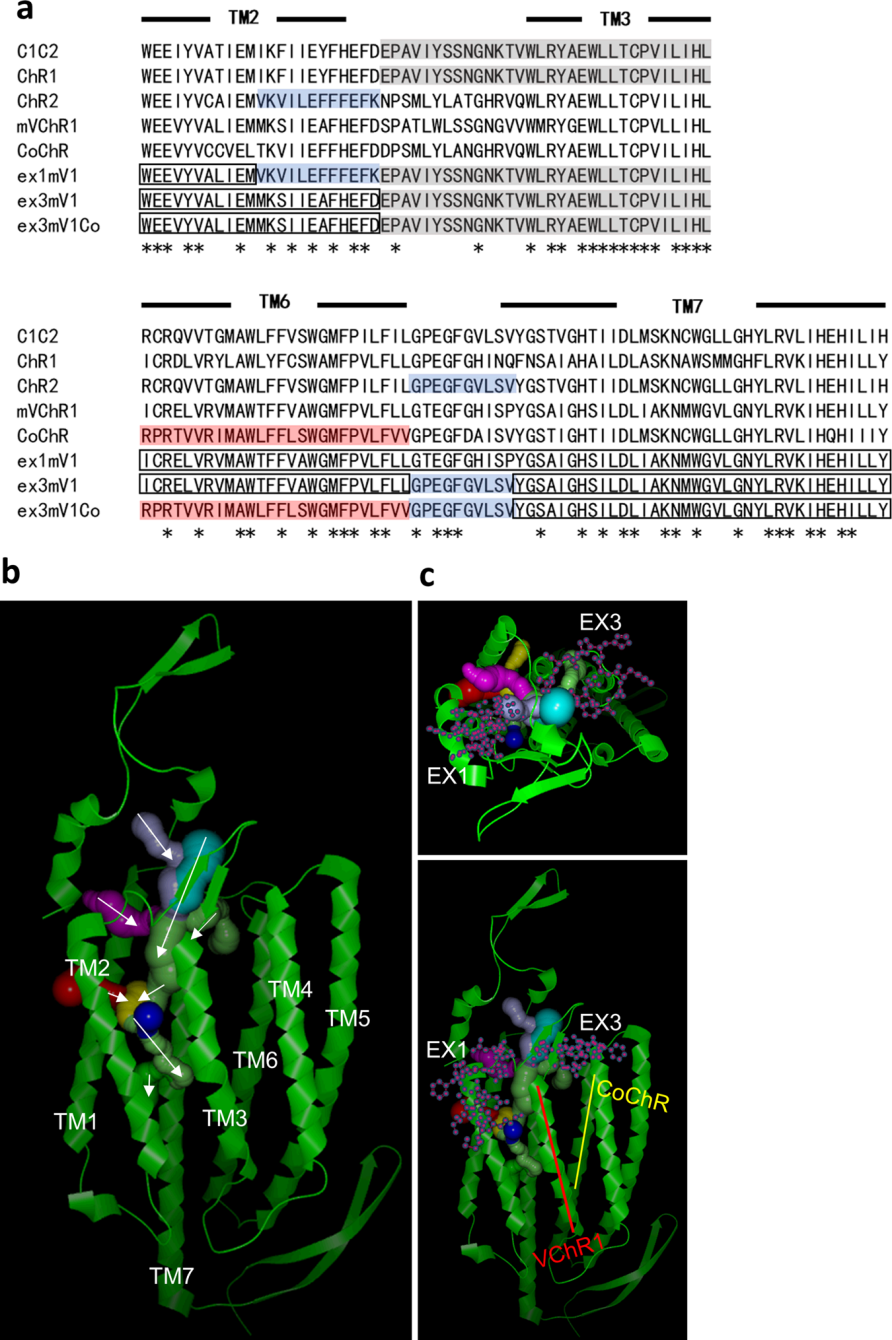
Nucleotide sequences encoding extracellular regions close to the simulated ion-conducting pathways in mVChR1 were replaced with the corresponding sequences of *Chlamydomonas*-derived channelrhodopsins and *Chloromonas oogama*-derived channelrhodopsin. **a** The amino acid sequences around the extracellular loops of TM2 and TM3 and TM6 and TM7 are shown. The replaced amino acids from channelrhodopsin-1, channelrhodopsin-2 and CoChR are shown as grey, blue and red background boxes, respectively. The open boxes indicate amino acids of mVChR1 that were not replaced. **b** The ion-conductive pathways were simulated by CAVER (http://www.caver.cz/). Some simulated tunnels are shown by white arrows. **c** The replaced amino acids and the transmembrane domain are indicated by purple dots and red and yellow bars. Extracellular regions between TM2 and TM3 and TM6 and TM7 are abbreviated as ex1 and ex3, respectively.

Ion-conducting pathways of ChR2 have been reported to be formed in TM1–3 and 7 structures^21^. The photocurrents of mVChR1, derived from VChR1, were lower than those of ChR2^16^. The ion-conductive pathways of mVChR1 were simulated based on the crystal structure of ChR2^21^. Our previous research investigating the photocurrent properties of the chimeric protein of ChR1 with ChR2 showed that the chimaera including TM3 of ChR1 increased its photocurrents (Fig. 1a, grey-background box). We expected the TM3 of ChR1 to play a role in increased photocurrent elicitation, based on our previous research^22^. The TM3 of mVChR1 was replaced with the corresponding sequence of ChR1 because this sequence was included in the simulated ion-conductive pathways of mVChR1 (Fig. 1b). The extracellular loop between TM2 and TM3 (ex1) and the extracellular loop between TM6 and TM7 (ex3) (Fig. 1c) were also identified at the entrance of the ion-conductive pathways.

### Photocurrents and kinetic profiles in three mutants

**Fig. 2:**
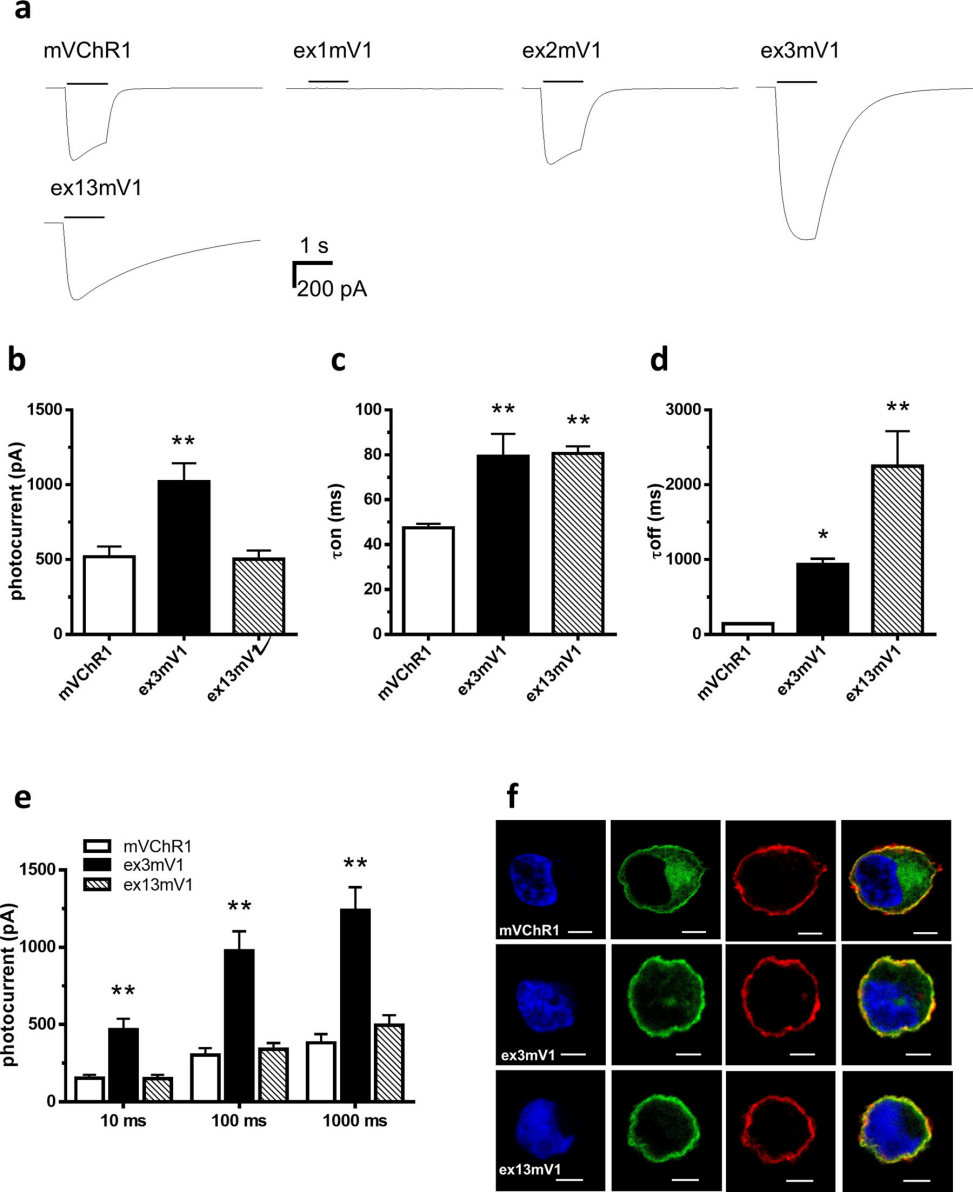
Kinetic profiles of photocurrents in cells expressing mVChR1, ex3mVChR1 and ex13mVChR1 genes. **a** Typical waveforms recorded from each gene-expressing cell with a stimulus of light at 505 nm for 1000 ms at 1 μW/mm^2^. **b** Comparison of peak photocurrents stimulated at 505 nm for 10 s. **c**, **d** Differences in the turning-ON (τON; c) and turning-OFF constants (τOFF; d) in cells expressing different genes, with a stimulus of light at 505 nm for 1000 ms at 1 μW/mm^2^. **e** Photocurrents elicited using various durations of light stimulus with 505 nm at 1 μW/mm^2^. **f** There was no difference between the protein localisation of each expressed gene. Bar = 5 μm. The numbers of mVChR1, ex3mVChR1 and ex13mVChR1 cells recorded in each graph from b to e were 11, 8 and 6, respectively. Data are presented as mean ± SEM (*p < 0.05, **p < 0.01; Dunnett’s multiple comparison test).

The photocurrents from HEK293 cells transfected with the mutant genes, ex3mV1 or ex13mV1, described in the Methods and Fig. 1, were compared with those from mVChR1-expressing cells. The amplitudes of the photocurrents from ex3mV1-HEK293 cells were significantly (*p* < 0.001) larger than those from mVChR1-HEK293 cells (Fig. 2b). The turning-ON (τON; Fig. 2c) and turning-OFF (τOFF; Fig. 2d) durations of ex3mV1- and ex13mV1-HEK293 cells were significantly longer than those of mVChR1-HEK293 cells. Analysis of the stimulus duration response revealed that the photocurrents of ex3mV1-HEK293 cells were larger for all stimulus durations compared to those of the mVChR1-expressing cells (Fig. 2e). In addition, the photocurrents of the cells transfected with ex2mV1, wherein the ex2 amino acid-encoding sequence was replaced with the corresponding one from ChR2, showed no significant difference compared with those of mVChR1-HEK293 cells (Fig. 2a and Supplementary Fig. 1). Venus fluorescence, indicating the expression of each gene, was found in similar locations within the HEK cells (Fig. 2f).

### Photocurrents in ex3mV1Co-expressing cells

**Fig. 3:**
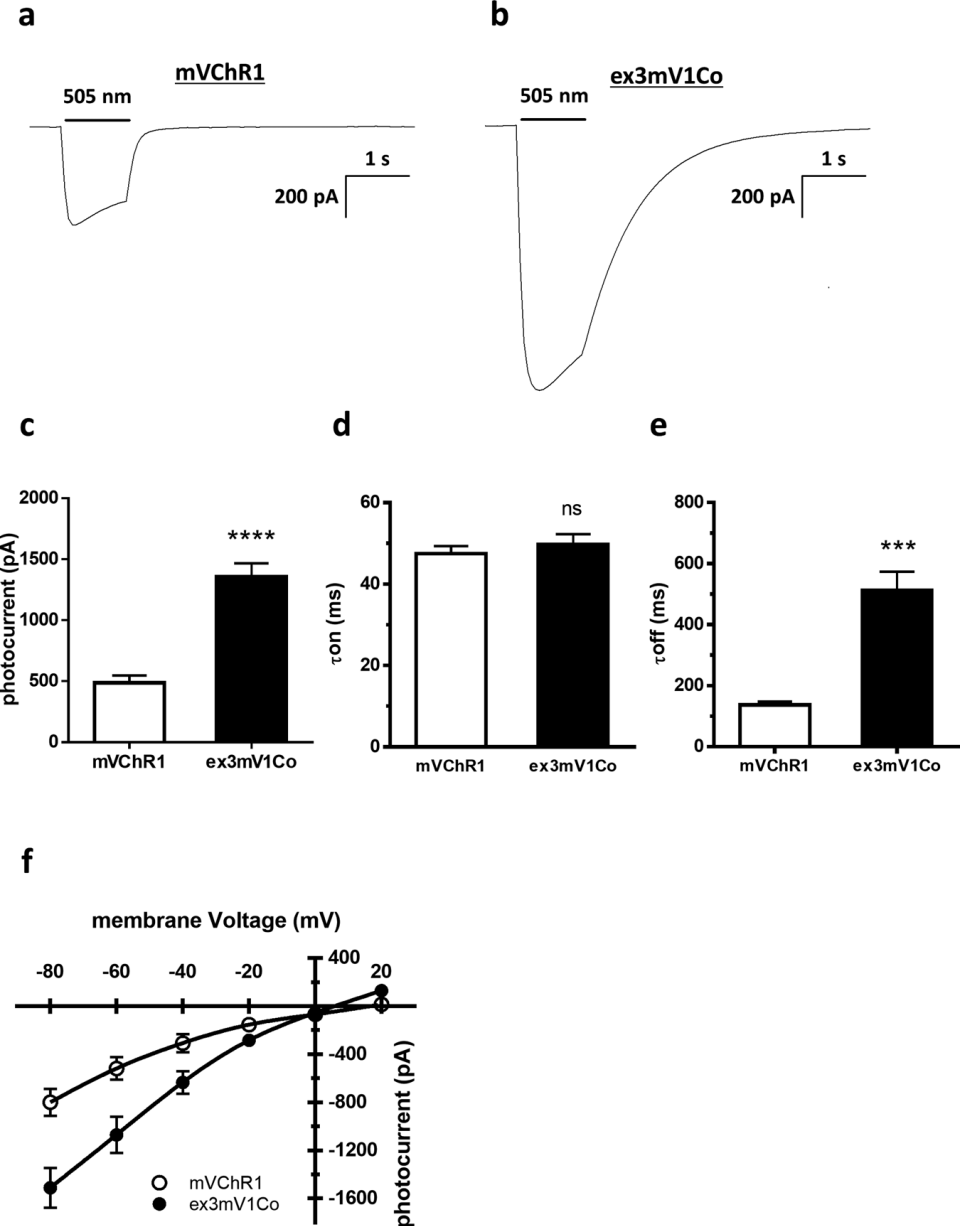
Comparison of kinetic profiles of photocurrents elicited from mVChR1- and ex3mV1Co- expressing cells. **a**, **b** Typical waveforms of cells expressing the mVChR1 or ex3mV1Co gene stimulated at 505 nm. **c** Comparison of peak photocurrents stimulated at 505 nm in mVChR1- and ex3mV1Co-expressing cells (mVChR1: *n* = 8, ex3mV1Co: *n* = 11). **d**, **e** Differences in the turning-ON and turning-OFF constants (τON; d, τOFF; **e**) in each cell with a light stimulus of 505 nm (mVChR1: *n* = 8, ex3mV1Co: *n* = 11). **f** *I*–*V* curves of mVChR1 and ex3mV1Co are presented (mVChR1: *n* = 6, ex3mV1Co: *n* = 5). The stimulus intensities for all recordings were applied for 1 μW/mm^2^, and the stimulus durations were set at 1 s, except for the τON- and τOFF-analyses. The stimulus duration of the τON- and τOFF-analyses were set at 10 s. Data are presented as the mean ± SEM (***p < 0.001, ****p < 0.0001; unpaired *t* test).

The photocurrent of ex3mV1Co, which was obtained by modifying TM6 and the C-terminal of ex3mV1, was also investigated. Typical waveforms for this photocurrent are shown in Fig. 3a, b. The photocurrent of ex3mV1Co was significantly larger than that of mVChR1 (Fig. 3c), and there was no significant difference in the τON between mVChR1-HEK293 cells and ex3mV1Co-HEK293 cells (Fig. 3d). The τOFF of ex3mV1Co-HEK293 cells was longer than that of mVChR1-HEK293 cells (Fig. 3e). Comparison of ex3mV1-HEK293 cells (Fig. 2a–c) and ex3mV1Co-HEK293 cells (Fig. 3c–e) showed that the replacement of TM6 and the C-terminal region affected the τON of ex3mV1. There were a slight difference in the current–voltage (*I*–*V*) curves between mVChR1-HEK293 and ex3mV1Co-HEK293 cells (Fig. 3f). The reversal potential (*x*-axis intersection) of ex3mV1Co-HEK293 cells was slightly shifted toward 0 mV, compared to that of mVChR1-HEK293 cells.

**Fig. 4:**
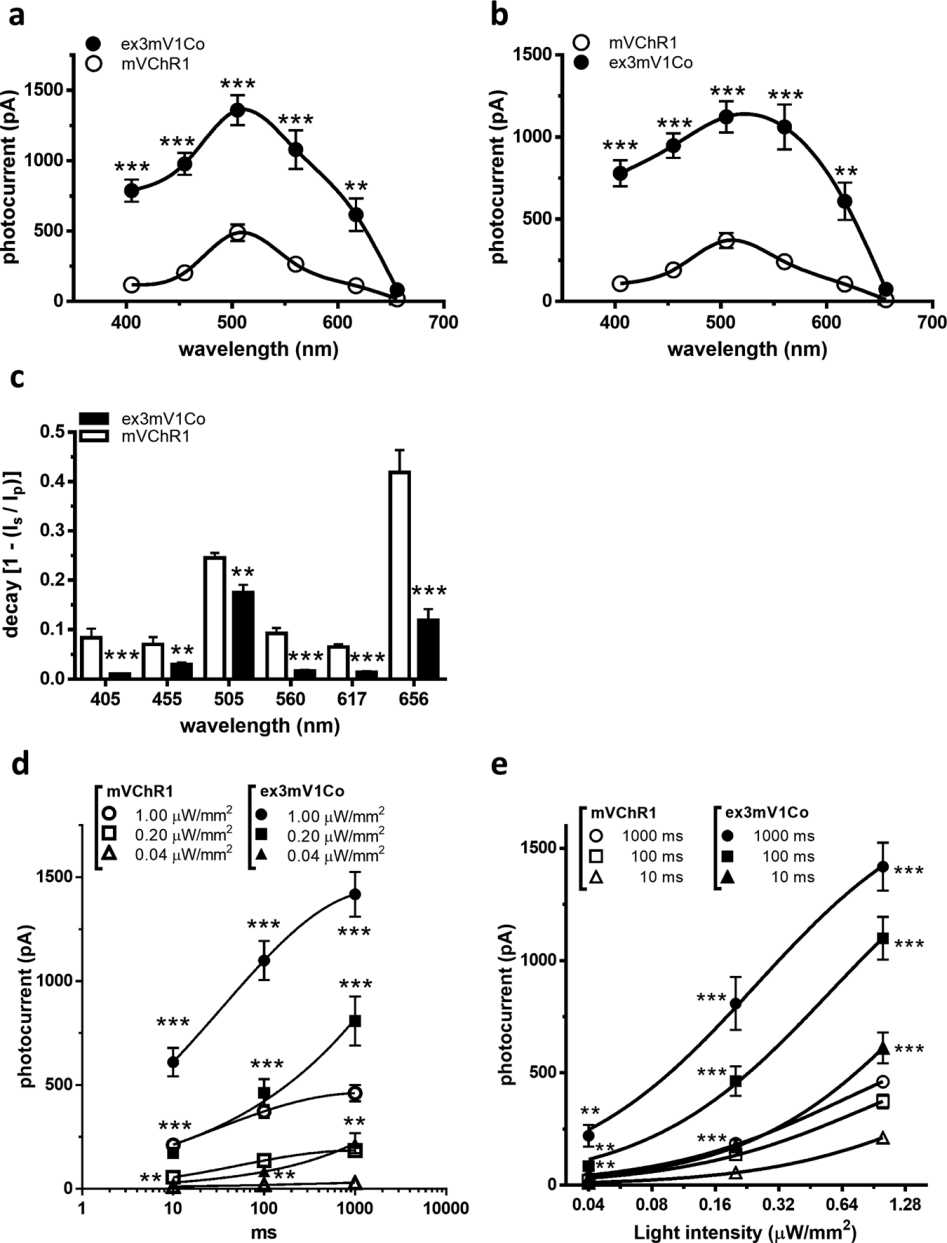
Wavelength sensitivity and intensity responses in mVChR1- and ex3mV1Co-expressing cells. **a**, **b** Each peak (**a**) and light-off photocurrent (**b**) were measured under the exposure of various wavelengths of light (405, 455, 505, 560, 617 and 656 nm) for 1 s (mVChR1: *n* = 8, ex3mV1Co: *n* = 11; *p < 0.05, **p < 0.01, ***p < 0.001; Tukey’s multiple comparison test). **c** Decay rates calculated from the data of the peak and light-off photocurrents. Data are presented as mean ± SEM (*p < 0.05, **p < 0.01, ***p < 0.001, unpaired *t* test). **d** Photocurrents were recorded for various stimulus intensities (0.04, 0.20 and 1.00 μW/mm^2^) and durations (10, 100 and 1000 ms) (*n* = 8–9) with LEDs of 505 nm.

We also investigated the wavelength sensitivities of ex3mV1Co. The peak photocurrents of mVChR1-HEK293 cells and ex3mV1Co-HEK293 cells were maximum at 505 nm, and the peak (Fig. 4a, *p* < 0.0001) and light-off photocurrents (Fig. 4b, *p* < 0.0001) of ex3mV1Co-HEK293 cells were significantly larger than those of mVChR1-HEK293 at 405–617 nm. The rates of decay of ex3mV1Co-HEK293 cells were significantly lower than those of mVChR1-HEK293 cells (Fig. 4c) at 405–656 nm. The decay rate of mVChR1-HEK293 cells was maximum at 505 nm, similar to that of ex3mV1Co-HEK293 cells. The light intensity responses and stimulus duration dependencies are shown in Fig. 4d, e, respectively. Even at light stimulus intensities of 0.04 μW/mm^2^, a photocurrent was clearly elicited from ex3mVC1Co-HEK293 cells. At a stimulus intensity of 0.2 μW/mm^2^, the photocurrent elicited from ex3mV1Co-HEK293 cells for a 10 or 100 ms stimulus duration was almost the same as that elicited from mVChR1-HEK293 cells with a stimulus intensity of 1.00 μW/mm^2^ (Fig. 4d). Moreover, with an increase in stimulus duration, the difference in the photocurrents between ex3mV1Co-HEK293 and mVChR1-HEK293 cells became larger. The slope of ex3mV1Co-HEK293 cells was significantly steeper than that of mVChR1-HEK293 cells at 10–1000 ms (Fig. 4e, one-way ANCOVA test, *p* < 0.001). All comparisons between mVChR1-HEK293 and ex3mV1Co-293 cells revealed statistically significant differences (*p* < 0.01).

### Adeno-associated virus-mediated ex3mV1Co gene transfection into genetically blind rats

**Fig. 5:**
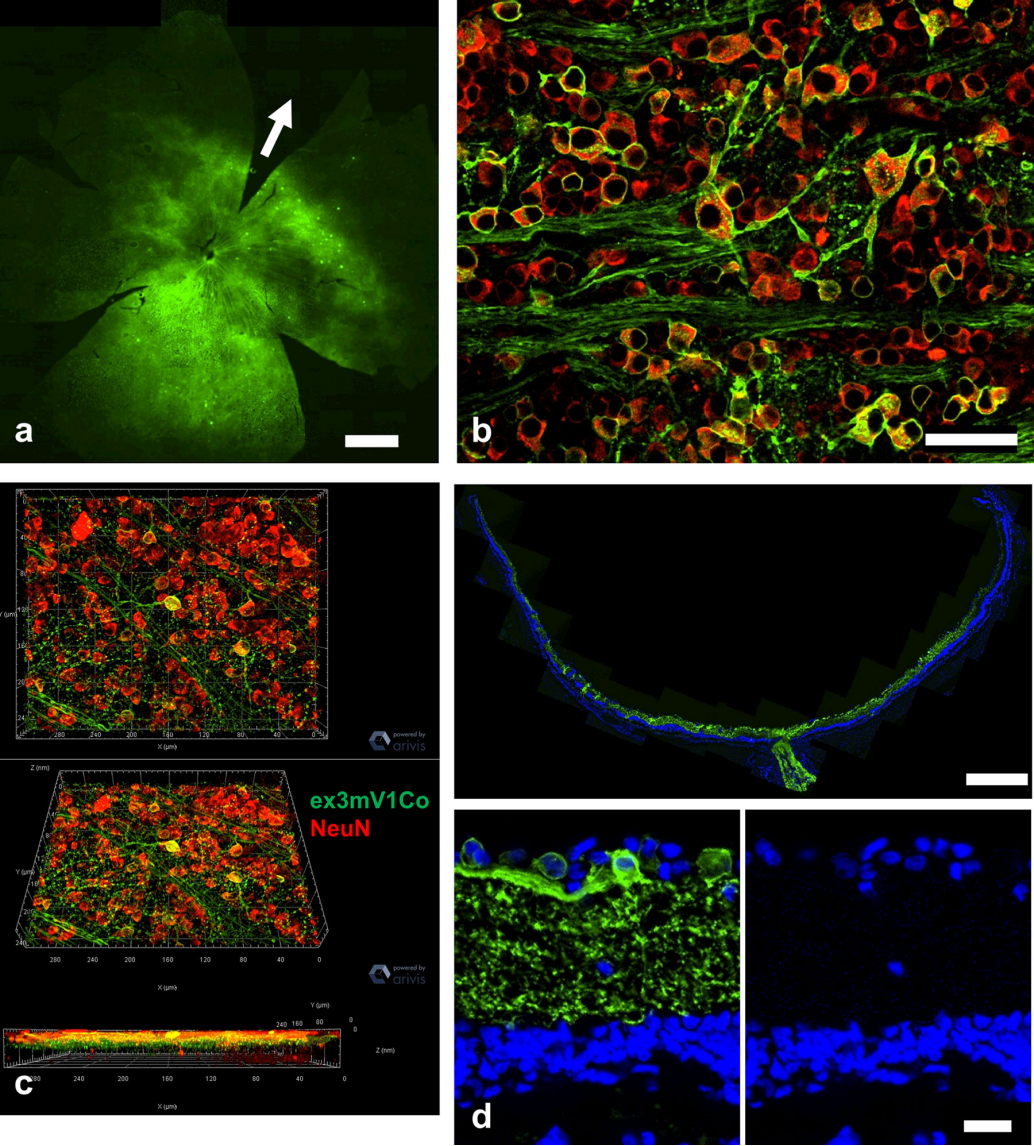
AAV-mediated ex3mV1Co gene transfection into blind rat retinas. **a** Venus fluorescence (green) in the whole-mounted retina. The arrow indicates the 12 o’clock direction. Bar = 1000 μm. **b** The retinal ganglion cells of whole-mounted retinas were identified by immunostaining with the anti-NeuN antibody (red). Bar = 50 μm. **c** The 3-D view of the immunostained retinas. **d** Cryo-retinal section. Nuclear staining (blue) was performed using mounting media, including 4′,6-diamidino-2-phenylindole (DAPI). Bar = 500 μm (upper), 20 μm (lower).

Adeno-associated virus (AAV)-mediated transfection of ex3mV1Co resulted in Venus fluorescence in the entire retina (Fig. 5a); however, the inferior part of the retina seemed to show better transfection efficiency. Most of the Venus fluorescence overlapped with rhodamine, indicating NeuN immunoreactivity (Fig. 5b). The 3-D image also showed co-labelling in an area on the retinal surface (Fig. 5c). The vertical section clearly indicated that ex3mV1Co was mainly expressed in the RGCs and the inner plexiform layer (Fig. 5d).

### Recording of visually evoked potentials (VEP)

**Fig. 6:**
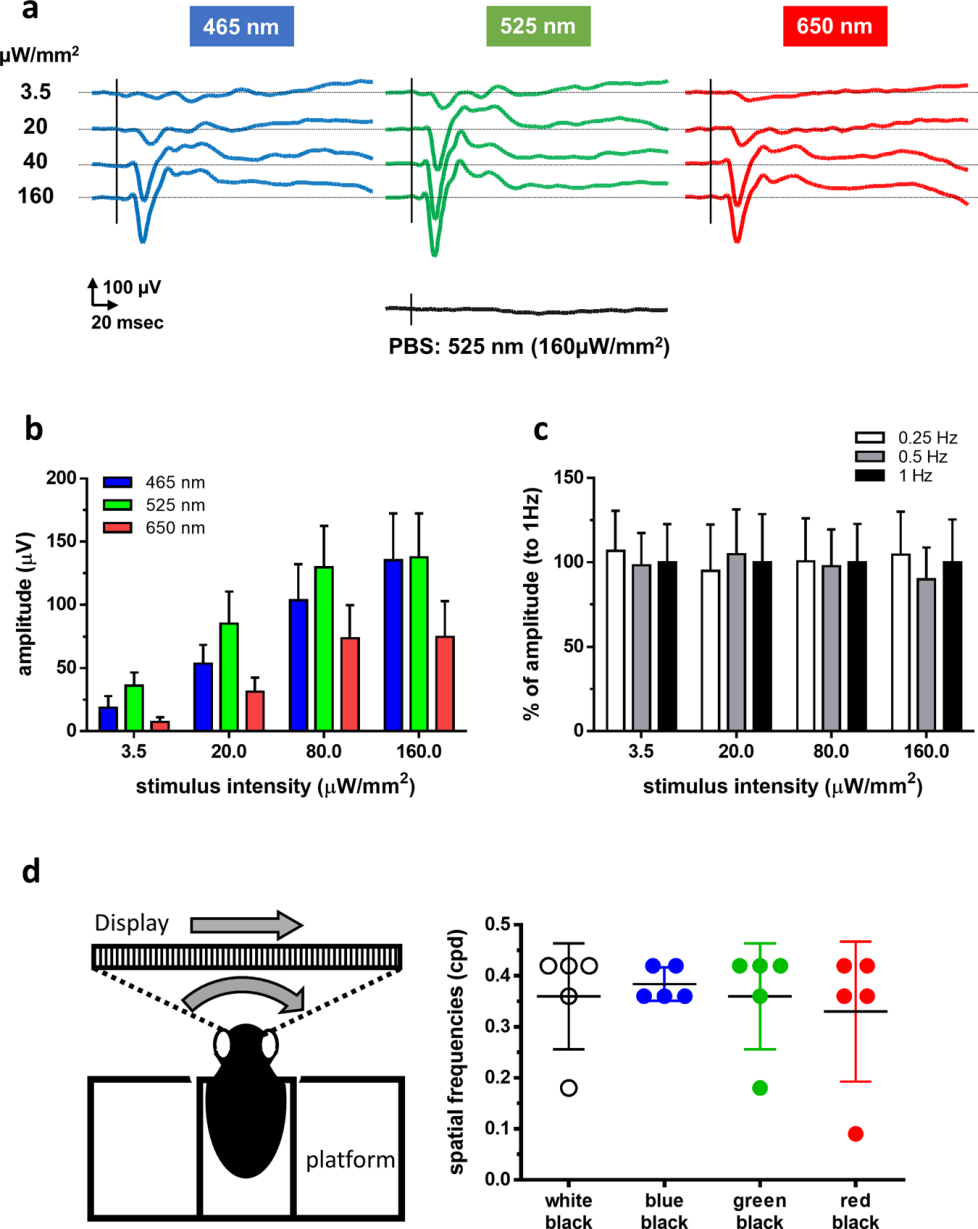
Recordings of VEPs and behavioural assessments in ex3mV1Co-transfected rats. **a** Typical waveforms of VEPs with stimuli at blue (465 nm), green (525 nm) and red (650 nm) LEDs. **b** Evoked potentials in response to each LED with various intensities (*n* = 8). **c** Effects of stimulus frequencies on the evoked potentials (*n* = 7). Data are presented as mean ± SEM. **d** Optomotor responses against white-black, blue-black, green-black and red-black stripes with various frequencies displayed on the screen (*n* = 5). Data are presented as mean ± SD.

Royal College of Surgeons (RCS) rats (age, 7–10 months), which are genetically blind, were used for the experiments; they showed no response in visually evoked potentials (VEPs) (Fig. 6a). However, 2 months after transfection of the ex3mV1Co gene, VEPs could be clearly recorded with stimuli of 465, 525 and 650 nm (Fig. 6a, b), even with a stimulus intensity of 3.5 μW/mm^2^. VEPs could be recorded even 17 months after the transfection, indicating the maintenance of the recovered visual function for a long duration (Supplementary Figure 2). We also measured the response to light flashes repeated at different frequencies (Fig. 6c), which indicated no decrease in responses.

### Behavioural tests

All of the ex3mV1CO-expressing rats were tracked with the range of spatial frequencies from 0.09 to 0.42 at the blue-black stripes (Fig. 6d). However, one among five rats could not track the spatial frequency at 0.36 of white-black or green-black stripes. In addition, one among five rats could not track the spatial frequency at 0.18 of red-black stripes.

## Discussion

In this study, we modified the *mVChR1* gene that we had previously synthesised using a bioinformatics approach, focusing on the ion-conductive pathways to improve light sensitivity. We found that the extracellular loop between TM6 and TM7 and TM6 play an important role in the photocurrent amplitude and channel kinetics, respectively. We also demonstrated that blind rats transfected with the ex3mV1Co gene could respond to irradiances measuring μW/mm^2^.

The replacement of ex1 unexpectedly resulted in a current loss in ex1mV1-expressing cells (Fig. 2a), whereas the replacement of ex3 produced a marked increase in current amplitude in ex1mV1-expressing cells (Fig. 2b). Surprisingly, ex13mV1, including both ex1 and ex3, produced photocurrents with amplitudes similar to those of mVChR1. The representatives of channel kinetics, such as the rates of τON and τOFF, were reduced for ex3mV1 and ex13mV1 compared to those for mVChR1 (Fig. 2c, d). These results indicated that ex1 and ex3 (Fig. 1) played an important role in the light-induced ion channel activities of mVChR1. The contributions of ex1 and ex3 to the photocurrent and channel kinetics are still unclear, but the fact that the loss of function caused by the replacement of ex1 was restored by the additional replacement of ex3 might indicate that the interactions between ex1 and neighbouring amino acids are related to its channel function.

To improve the channel kinetics, various mutants, such as chimeric channelrhodopsins and channelrhodopsin with single amino acid substitutions, have been reported^23^. In addition, the 3-D structure of the protein has been reported to affect its interaction with nearby transmembrane domains, which subsequently affects the channel characteristics^24^. We additionally modified ex3mV1 to improve the kinetics (such as τON and τOFF) of ex3mV1. CoChR has been reported to have fast kinetics and good membrane trafficking^25,26^. Ganjawala et al.^27^ also reported some positions of amino acid residues having an important role in the kinetics of the off-rate in CoChR. Meanwhile, we focused on the TM6 of CoChR, which is expected to affect the CoChR protein structure owing to the presence of retinal binding sites (corresponding to W261 of mVChR1) and was introduced into ex3mV1. Although the τON and τOFF rates of ex3mV1Co became shorter than those of ex3mV1, the τOFF of ex3mV1Co was not as short as that of mVChR1 (Figs. 2b, c and 3d, e); the mechanism underlying this phenomenon remains unclear. The molecular basis of photocurrent kinetic profiles has been explained using multiple-photocycle models^28,29^ defined as the transition rates, such as the closing state and the opening state^30,31^. In the present study, we could not elucidate the photocycle because of the use of the whole-cell patch–clamp. Further studies using single-channel recordings such as the patch–clamp methods of cell-attached, inside-out or outside-out mode are needed to clarify the photocycle model of ex3mV1Co.

The peak photocurrent of ex3mV1Co was increased compared to that of ex3mV1, and the opening speed of ex3mV1Co remained the same as that of mVChR1, but the closing speed of ex3mV1Co was slower than that of mVChR1. The difference in the primary amino acid structures between ex3mV1 and ex3mVCo is mainly TM6. The position of proline^246^ in the TM6 of ex3mV1Co—the corresponding amino acid of which is cysteine^246^ in ex3mV1—is related to the ion-conductive pathway and forms the ion tunnel with valine^241^ in TM5 and isoleucine^309^ in TM7, as revealed by a CAVER simulation (Supplementary Fig. 3a, b). The diameters of the ion tunnel at position 246 in ex3mV1Co and ex3mV1 were 1.33 and 1.42 Å, respectively. This difference in diameters did not seem to result in a difference in kinetics. The average distance between the amino acid at position 246 and I^309^ of ex3mV1Co was longer than that between the amino acid at position 246 and I^309^ of ex3mV1, although the average distance between the amino acid at position 246 and valine^241^ was almost the same in the molecular dynamics simulation (Supplementary Fig. 4a). The longer distance between the amino acid at position 246 and I^309^ of ex3mV1Co, owing to the replacement of TM6 [cysteine^246^(ex3mV1) to proline^246^(ex3mV1Co)], is expected to be one of the factors contributing to its increased photocurrent. In addition, the average potential energy between the amino acid at position 246 and I^309^ of ex3mV1Co was lower than that of ex3mV1 (Supplementary Fig. 4b). The faster opening kinetics of ex3mV1Co than that of ex3mV1 might be due to the weaker interaction of proline^246^ with isoleucine^309^ in ex3mV1Co than that in ex3mV1, and its decreased potential energy might affect its photosensitivity.

The reversal potential, one of the indications for the ion selectivity, of ex3mV1Co has shifted about 15 mV to the negative direction compared to that of mVChR1. The equilibrium potential of sodium ions is approximately +60 mV. The reversal potential is close to +60 mV for highly sodium ion-selective channels^32^. In addition, the reversal potential shifts in the negative direction in channels when other cations, such as protons and potassium ions, are also permeable^33,34^. The negative shift of the reversal potential may be due to a change in ion selectivity—for example, a lower sodium-selectivity in ex3mV1Co than in mVChR1.

In cation channelrhodopsin, ChR2, the amino acid residues in the transmembrane domain has an important role in ion selectivity^35,36^. There is no report that the key residue related to the ion selectivity is in the extracellular loop. However, in anion channelrhodopsin, the amino acid residue R84, the extracellular-loop amino acid residue R84 helps determine anion permeability^37,38^. Therefore, our data indicated that amino acids at ex3, the extracellular loop located between TM6 and TM7 of ex3mV1Co, in ex3mV1Co might be involved in sodium-ion selectivity.

Gene therapy using ChRs that target surviving neurons, RGCs, or bipolar cells is expected to be a promising strategy for restoring vision to blind patients. We aimed to determine whether ex3mV1Co can function in a rat model of retinal degeneration. It was confirmed that ex3mV1Co-Venus was expressed in vivo and that the maximum photosensitivity of ex3mV1Co was observed in the presence of green light, as observed in vitro. In addition, the developed ex3mV1Co exhibited broader wavelength sensitivities towards blue, green, and red light (Fig. 6). Therefore, ex3mV1Co can also function in vivo. More importantly, rats transfected with the ex3mV1Co gene acquired the function of responding to light in the order of μW/mm^2^. Behavioural studies also demonstrated that rats could respond to moving stripes presented on a liquid crystal display, although the responses differed with the colour displayed. The recovered visual function obtained by gene therapy would likely be highly dependent on the gene transfection into the retina^39^. Therefore, transfection of ex3mV1Co could be a potent technique to restore advanced visual function. However, a well-organised visual system like human vision recognises even a single photon^40^. The threshold response to light in ex3mV1Co was 0.04 µW/mm^2^ (Fig. 4e), which corresponds to approximately 10^11^ photons/mm^2^. Thus, even though ex3mV1Co shows remarkably high light sensitivity compared to other optogenetic proteins, its light sensitivity is still considerably different from that of natural vision. Therefore, there might be a need to use a combination of other mechanical devices such as a goggle apparatus or other optogenetic genes with ex3mV1Co-gene therapy so that patients might achieve visual function comparable to that of natural vision.

We found that the third extracellular loop was implicated in the selectivity of sodium ions and that the TM6 and C-terminal regions played some roles in channel kinetics. Our findings contribute to the development of novel optogenetic genes that may be more sensitive to light and exhibit different kinetic profiles. However, currently, there is no information that might allow the control of wavelength sensitivities. Further study is needed to identify the amino acid sequences that determine wavelength sensitivities.

## Methods

### Design of mutants and plasmid preparations

To improve ion permeability, TM3 and the nucleotide sequences encoding three sites (ex1, ex2 and ex3) close to the extracellular loops in mVChR1 were replaced with those encoding the corresponding sites of ChR1 and ChR2, respectively. TM3 of ChR1 (Fig. 1a) was expected to contain the amino acid sequence related to conductance^22^, and the ex1 and ex3 sites were considered to be associated with the ion-conducting pathways, as indicated by the CAVER 3.0 simulation^41^ (Fig. 1b) based on the previously reported crystal structure of C1C2^42^. The genes encoding the amino acid sequences of the ex1 and ex3 sites in mVChR1 were replaced with the corresponding sequences in ChR2 and named ex1mV1 and ex3mV1, respectively, and the gene containing replacements for nucleotide sequences encoding both sites was named ex13mV1 (Fig. 1c). Further, the sequence encoding TM6 and the C-terminal region of ex3mV1 was replaced with the corresponding sequence of *Chloromonas oogama*-derived channelrhodopsin (CoChR)^43^ to form ex3mV1Co.

The plasmid vectors containing each gene were designed as previously described^44^. In brief, the N-terminal fragment of each gene was fused to Venus fluorescent protein in frame at the end of each gene-coding fragment. The Venus-fused cDNAs were then subcloned into the *Eco*RI and *Bam*HI sites of the 6P1-CAG plasmid^12^. These plasmid vectors were used for transfecting the respective genes into HEK293 cells and for preparing the AAV type 2 vector.

### Cell preparation

HEK293 cells (RCB1637: Riken Bioresource Center, Tsukuba, Japan) were cultured in a minimum essential medium (Thermo Fisher Scientific, Tokyo, Japan) supplemented with 10% foetal bovine serum under a 5% CO_2_ atmosphere at 37 °C. The culture medium was changed every 3 days, and cells were passaged at 80% confluence using a 0.02% ethylenediaminetetraacetic acid (Sigma-Aldrich, St. Louis, MO)/DPBS solution.

### Gene transfection into HEK293 cells

HEK293 cells were transfected with the pAAV type 2 plasmid vectors (Agilent Technology, Tokyo, Japan) containing a CAG promoter using a slight modification of the calcium phosphate method^45^. In brief, cells were seeded onto the 60 mm culture dish at a concentration of 2.5 × 10^6^ cells 1 day before the transfection, and the culture medium was replaced before transfection. The calcium phosphate-DNA particles using 6 μg of plasmid DNA were then added to the 60 mm dish, and the medium was replaced with fresh culture medium following incubation in a 5% CO_2_ atmosphere at 37 °C for 6 h. The cells were incubated in a 5% CO_2_ atmosphere at 37 °C for 24–30 h and were then seeded onto glass slides 1 day before the patch–clamp recordings were performed.

### Patch–clamp recordings

Whole-cell patch–clamp recordings were performed using cultured HEK293 cells 2 days post transfection. Photocurrents were elicited by stimuli of various wavelengths (405, 455, 505, 560, 617 and 656 nm) from light-emitting diodes (Mightex Systems, Pleasanton, CA, USA) and recorded using an EPC-10 amplifier (HEKA Electronik, Lambrecht, Germany) in whole-cell patch–clamp mode. The intensity of light at each wavelength was adjusted to 1 µW/mm^2^ by setting an appropriate current for each wavelength. The data were collected by filtering at 10 kHz and sampling at 5 kHz. The photocurrent was measured as the increase in inward current, and series resistance compensation was applied at 70%. The internal solution contained 130 mM CsCl, 10 mM HEPES, 2 mM MgCl_2_, 0.1 mM CaCl_2_, 10 mM NaCl, 2 mM Na_2_ATP and 1.1 mM EGTA, with the pH adjusted to 7.2. The external solution contained 138 mM NaCl, 3 mM KCl, 1 mM CaCl_2_, 2 mM MgCl_2_, 4 mM NaOH and 10 mM HEPES, with the pH adjusted to 7.4 by HCl. The τON and τOFF time constants were analysed as previously described^22,46^ (Supplementary Fig. 5). In brief, τON was measured as the time to reach 1 − e^−1^ (63%) of the peak amplitude at the maximal photocurrent during exposure to the light stimulus. The τOFF was measured as the time to reach e^−1^ (37%) of the light-off amplitude at the end of exposure to the light stimulus. The decay was quantified as the difference between the peak and light-off amplitudes divided by the peak amplitude.

### Animals

All animal experiments were conducted in accordance with the guidelines proposed by the Animal Care and Use Committee of Iwate University, Japan (approval No. A201801). The experiments were conducted on 7–10-month-old Royal College of Surgeons (RCS; rdy/rdy)^47^ rats obtained from CLEA Japan (Tokyo, Japan). The rats were housed under conditions of cyclic light (On: 8:00 AM, 200 lux; Off: 8:00 PM) at 23 °C ± 1 °C and provided ad libitum access to laboratory chow and water. The average weights of the rats were 250.686 g (Fig. 6b), 278.5 g (Fig. 6c) and 535.44 g (Fig. 6d).

### Preparation of the AAV vector

Plasmid vectors containing ex3mV1Co were used to produce the AAV type 2 vector. The AAV Helper-Free System (Agilent Technologies, Santa Clara, CA, USA) was used to produce infectious AAV virions, according to the manufacturer’s instructions. AAV vectors were purified using a previously described the single-step column purification method^48^. The concentration of the purified AAV vectors was determined by measuring the levels of the AAV capsid protein using ELISA (PROGEN, Heidelberg, Germany).

### Intravitreal injection of AAV

Rats were anaesthetised by intramuscular injection of 35 mg kg^−1^ ketamine and 3.5 mg kg^−1^ xylazine. Under an operating microscope, 5 μL of a suspension containing 1 × 10^12^ virions mL^−1^ was intravitreally injected through the ora Serrata using an automatic syringe (Neurosyringe AC; ACrux Inc., Morioka, Japan) with a 32-gauge needle (Hamilton Company, Reno, NV, USA).

### Recordings of VEPs

Two months after the intravitreous injections, VEPs were recorded as described previously^16^. Briefly, at least seven days before the recordings, the electrodes were implanted epidurally 6.8 mm behind the bregma and 3 mm lateral to the midline of both hemispheres. A reference electrode was implanted epidurally on the midline 12 mm posterior to the bregma. VEPs were recorded using a PuREC (Mayo Corp., Aichi, Japan). First, the rats were anaesthetized with intramuscular ketamine (35 mg/kg) and xylazine (3.5 mg/kg), and then, their pupils were dilated with 0.5% tropicamide and 0.5% phenylephrine hydrochloride. Photic stimuli of various intensities were generated using different colour (wavelengths: 468, 525 and 640 nm) LEDs (VE-LED; ACrux Inc.) and applied for 10 ms with a frequency of 1 Hz. The high- and low-pass filters were set at 3 and 500 Hz, respectively. VEP responses were consecutively measured 200 times, and the collected response waveforms were averaged.

### Preparation of retinal sections and whole-mounted retinas

Rat eyes were fixed overnight at 4 °C in 4% paraformaldehyde prepared in phosphate-buffered saline (PBS; Fujifilm Wako Pure Chemical, Osaka). The eyes were rinsed with PBS, and the anterior parts, such as the cornea, iris, and lens, were removed. For preparing retinal sections, the eyecups were immersed in 10%, 20% and 30% sucrose in PBS and embedded in optimal cutting temperature compound (Sakura, Tokyo, Japan) using a Histotech Pino^®^ system (Sakura, Tokyo, Japan). Cryosections (12 μm) of the tissue were mounted on slides and air-dried. Whole-mounted retinal specimens were prepared by detaching the retinas from the eyecups and immunostaining them with an anti-NeuN antibody (MAB377; Merk Millipore, Tokyo, Japan).

### Immunohistochemistry

Retinal sections were washed with PBS containing 0.3% Triton X-100 (0.3% Tx-PBS) and treated for 10 min with 3% Tx-PBS. Sections were blocked with 3% normal goat serum (Funakoshi, Tokyo) and 1% BSA in 0.3% Tx-PBS for 30 min at room temperature (20–25 °C) and incubated with ALEXA FLUOR^®^ 594-conjugated anti-NeuN polyclonal antibody (2 μg/mL; Cat No. bs-1613R-A594, Bioss, MA, USA) overnight in a moist chamber at 4 °C. After the slides were washed, coverslips were mounted with DAPI fluoromount-G^®^ (Cosmo-Bio, Tokyo, Japan). Retinas were treated with hyaluronidase (300 units/mL; Cat No. H3884, Merk, Tokyo, Japan) for 2 h at room temperature. After the retinas were washed with 0.5% Triton X-100 prepared in PBS (0.5% Tx-PBS), they were incubated with ALEXA FLUOR^®^ 594-conjugated anti-NeuN polyclonal antibody (5 μg/mL) diluted in PBS containing 2% normal goat serum and 2% Triton X-100 overnight at room temperature. After retinas were washed with 0.5% Tx-PBS, they were flat-mounted on slides and covered with fluoromount-G^®^ (Cosmo-Bio). The sections and flat-mounted retinas were observed under a fluorescence microscope (Carl Zeiss, Tokyo, Japan).

### Behavioural assessment

The spatial vision of an ex3mV1CO-expressing rat was measured by its optomotor response. We used a virtual optomotor system that was produced according to a sine wave function with the variable frequency with a stimulus of colour stripes over a black background previously described^16^ with slight modification. We used a liquid crystal display instead of the projector that was previously used because ex3mV1Co has a higher sensitivity than mVChR1. The maximum intensity reached 103 lux of white light at the platform. The luminance values at the centre of the platform were 30, 40 and 36 lux when the colour was set to blue, green and red, respectively. Each wavelength displayed on the screen was measured by using an illumination metre (LX-1128SD; Mother Toll, Nagano, Japan). The experimenter waited until the rat stopped moving, and then, a homogeneous grey stimulus was projected for 5 s on the screen before the presentation of each grating session. The grating session was displayed with an alternative direction every 5 s, and the session was displayed for 60 s. An experimenter assessed whether the animals tracked the rotation by monitoring the head movement and presented the rotating stimulus simultaneously on the video camera monitor. If the movement was ambiguous, the same grating session was presented again. All behavioural tests were double-blinded and performed during the first few hours of the animals’ light cycle (lights on at 8:00 AM).

### Statistical analysis

Statistical analyses for in vitro experiments were performed using Sigma Plot ver. 12.3 (Hulinks, Tokyo, Japan) and MATLAB R2021a (The MathWorks, Natick, MA, USA). The statistical methods used were Tukey’s multiple comparison test, Dunnett’s multiple comparison test, Student’s *t* test, and one-way ANCOVA. A *p*-value < 0.05 was considered significant.

### Molecular dynamics simulations

The initial structural coordinates of ex3mV1 and ex3mV1Co proteins were determined based on the ground-state crystal structure of channelrhodopsin derived from PDB: 4yzi), using the homology modelling server SWISS-MODEL (https://swissmodel.expasy.org/). The model structure contains the protein dimer, covalently bound retinal chromophores, 5 oleic acids and 19 water molecules per protein monomer.

The potential energy function was set as follows: the consistent-valence forcefield force field was used for proteins and oleic acid, while the extensible and systematic force field ESFF was used for polyene chains (retinal). The following protocol was employed to perform an MD simulation: a simulation system was geometrically optimised by performing a 5000-step conjugate gradient energy minimisation. We fixed heavy atoms of proteins, as well as oleic acids and water molecules degraded in the crystal structure, during geometry optimisation. An equilibration calculation was performed by progressively decreasing position constraints in 200 ps MD simulations, starting from a force constant of 25–10 and 1 kcal/mol Å, respectively. A 1-ns simulation was then performed at a constant temperature of 298 K in the National Variety Trial ensemble, without the position constraint. An MD simulation was performed using the Discover/Insight II software package (Dassault Systemes BIOVIA, Discovery Studio Modelling Environment, Release, 2017, San Diego: Dassault Systemes, 2016). The time step was integrated at 1 fs, and the cut-off of the short-range interaction was 9.5 Å. The Discovery Studio 4.5 software package was used to manipulate molecular graphics and perform simulation analysis.

### Reporting summary

Further information on research design is available in the Nature Research Reporting Summary linked to this article.

## Supplementary information

Supplementary Materials

Reporting Summary

## Data Availability

All data supporting the findings of this study are available from the corresponding authors upon reasonable request.
