# Supplementary material for: Development of an optogenetic gene sensitive to daylight and its implications in vision restoration
Source: NPJ Regen Med. 2021 Oct 14;6:64. doi: 10.1038/s41536-021-00177-5 (PMC8516861; doi:10.1038/s41536-021-00177-5)

Supplementary Table 1 One-way ANOVA with Dunnett's method for the data of the peak, τon, and τoff at 505 nm of ex3mV1 and ex13mV1 compared to those of mVChR1 in Figure 2a–c

	p-value	
	ex3mV1	ex13mV1
Peak current	0.001	0.988
τon	0.002	0.003
τoff	0.02	<0.001

Supplementary Table 2 One-way ANOVA with Dunnett's method for the photocurrents recorded for various durations at 505 nm of ex3mV1 and ex13mV1 compared to those of mVChR1 in Figure 2d

	p-value	
	ex3mV1	ex13mV1
10 ms	0.003	0.999
100 ms	<0.001	0.971
1000 ms	<0.001	0.834

Supplementary Table 3 Unpaired t-test for the data of the peak, τon, and τoff at 505 nm between mVChR1 and ex3mV1Co in Figure 3c–e

	p-value
	505 nm
Peak	0.00000659
τon	0.525
τoff	0.0000668

Supplementary Table 4 Unpaired t-test for the data of the peak, steady photocurrents, and decay between mVChR1 and ex3mV1Co in Figure 4a–c

		Peak current	Steady current	Decay
p-value	405 nm	0.00000175	0.00000168	0.000173
	455 nm	0.00000026	0.000000225	0.00499
	505 nm	0.00000659	0.00000836	0.00321
	560 nm	0.00013	0.000114	0.000000448
	617 nm	0.00129	0.00125	0.000000103

	656 nm	0.000435	0.000346	0.00000658

Supplementary Table 5 Unpaired t-test for the photocurrents recorded for various stimulus intensities and durations between mVChR1 and ex3mV1Co in Figure 4d and e.

	p-value		
	10 ms	100 ms	1000 ms
0.04 μW	0.00813	0.00264	0.0016
0.20 μW	0.000844	0.000263	0.00013
1.00 μW	0.0000864	0.00000523	0.000000885

Supplementary Table 6 Unpaired t-test for the data of the peak, τon, and τoff at 505 nm between mVChR1 and ex2mV1, and the photocurrents recorded for various stimulus intensities and durations between mVChR1 and ex2mV1 in Figure S1 a–d

	p-value
Peak current	0.523
τon	0.105
τoff	0.00111

Stimulus durations	p-value
10 ms	0.0754
100 ms	0.0777
1000 ms	0.169

Supplementary Table 7 Slope analysis of induced photocurrents with the stimulus durations. One-way analysis of covariance was performed.

mVChR1: ex3mV1Co	p-value
10 ms	4.35943×10^{-7}
100 ms	9.59434×10^{-7}
1000 ms	0.0001

Supplementary Figure 1 Comparison of the kinetic profiles of photocurrents in mVChR1- and ex2mV1-expressing cells. (**a**) Comparison of peak photocurrents for 505 nm light stimulus in cells expressing mVChR1 and ex2mVChR1. (**b**) Differences in peak photocurrents elicited by various stimulus durations at the intensity of 1 μW/mm^2. (**c**, **d**) Differences in the turning-ON and turning-OFF constants (c: τON, d: τOFF) in each cell with a light stimulus of 505 nm for 10 s at an intensity of 1 μW/mm^2. Data are presented as mean ± SEM. (**p < 0.001, unpaired *t*-test, mVChR1: n = 9, ex2mVChR1: n = 4).

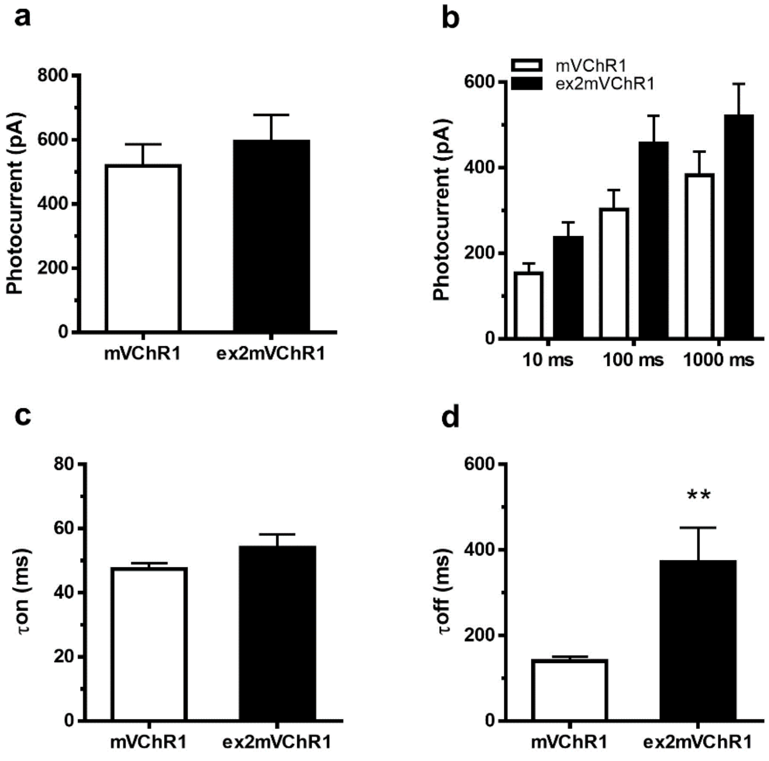

Supplementary Figure 2 Changes in visually evoked potentials in ex3mV1Co-expressing rats at 2, 5, and 12 months after AAV administration.

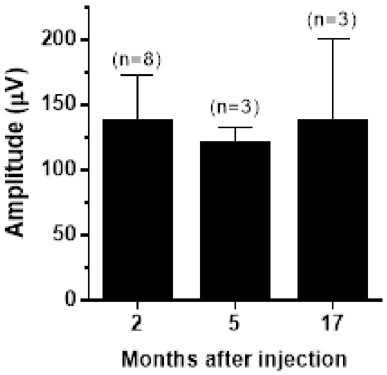

Supplementary Figure 3 Simulated ion tunnel at position 246

The Pro246 residue (ex3mV1Co; **a**)/Cys246(ex3mV1; **b**) forms an ion tunnel with Val241 in TM5 and Ile^{309} in TM7. The lengths of these are 1.33 and 1.42 Å, respectively.

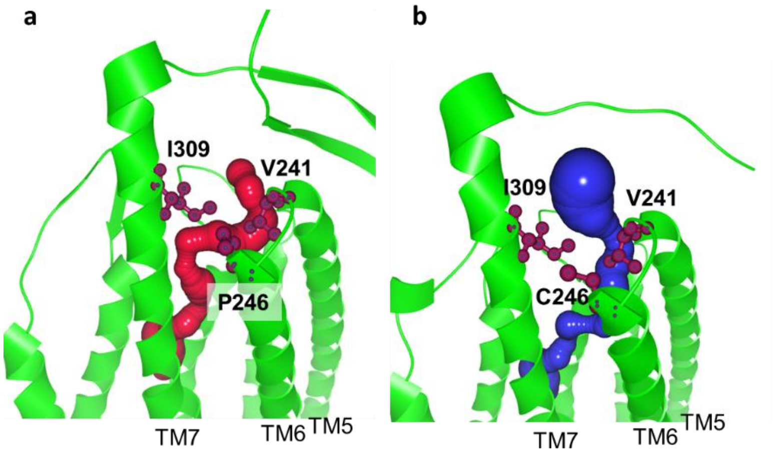

Supplementary Figure 4 Molecular dynamics simulations of ex3mV1 and ex3mV1Co

Molecular dynamics simulations were performed at 298 K. (**a**) The average distances between the amino acid residue at position 246 and Ile^{309} in ex3mV1Co and ex3mV1 were 8.3 and 6.9 Å, respectively. (**b**) The average distance between the amino acid residue at position 246 and Ile^{309} of ex3mV1Co was 1.4 Å longer than that of ex3mV1. The potential energies between the amino acid at position 246 and Ile^{309} in ex3mV1Co and ex3mV1 were −0.84 kcal/mol and −1.18 kcal/mol, respectively. The potential energy of ex3mV1Co was lower than that of ex3mV1.

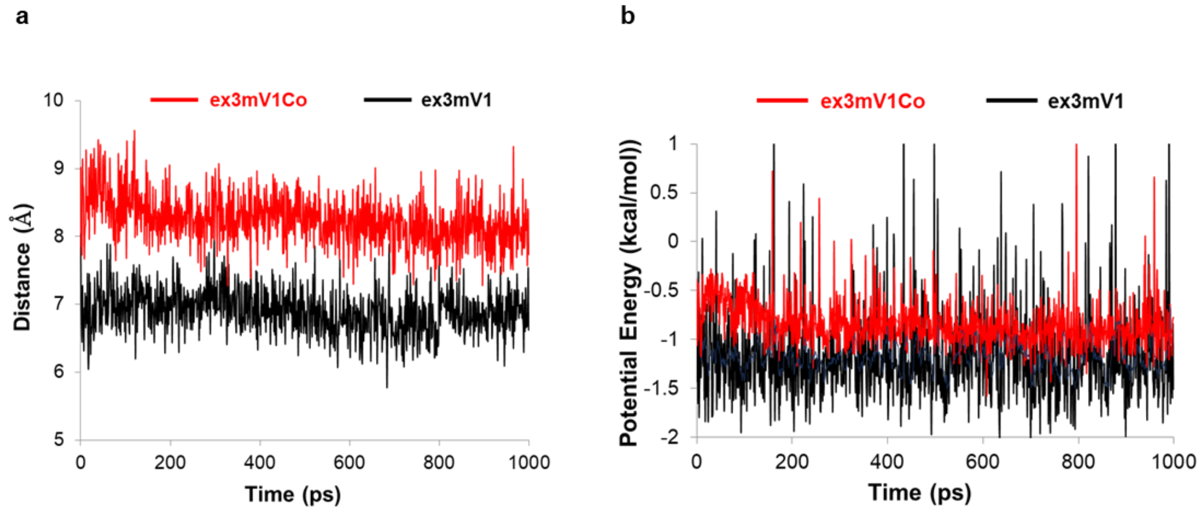

Supplementary Figure 5 Analysis of waveforms from patch-clamp data.

τon was the time to reach to about 63% of the peak amplitude from the time of the light on. τoff

was the time to reach to about 63% reduction from the light-off amplitude. Decay is shown as the value of the light-off amplitude divided by the peak amplitude subtracted from 1.

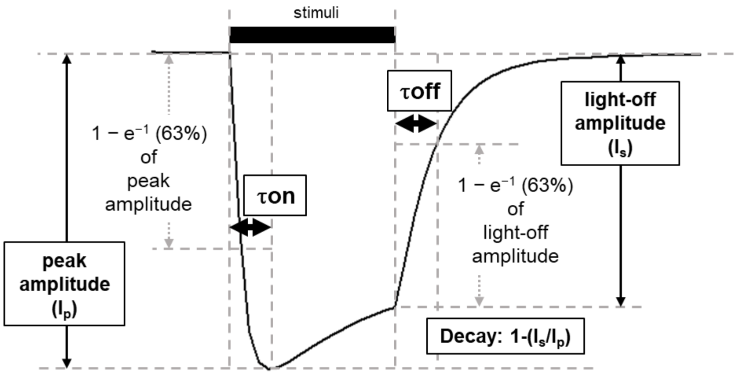

Supplement: Supplementary file 1 — Supplementary Materials [file 41536_2021_177_MOESM1_ESM.pdf]
